# Urinary metabolomic signature of esophageal cancer and Barrett’s esophagus

**DOI:** 10.1186/1477-7819-10-271

**Published:** 2012-12-15

**Authors:** Vanessa W Davis, Daniel E Schiller, Dean Eurich, Michael B Sawyer

**Affiliations:** 1 Department of Surgery, 2D2.01 Walter Mackenzie Health Sciences Center, University of Alberta, 8440 112th Street, Edmonton, Alberta, T6G 2B7, Canada; 2 School of Public Health, 2–040 Li Ka Shing Center for Health Research Innovation, University of Alberta, Edmonton, Alberta, T6G 2B7, Canada; 3 Cross Cancer Institute, 11560 University Avenue, Edmonton, Alberta, T6G 1Z2, Canada

**Keywords:** Metabolomics, Urine, Esophageal cancer, Barrett’s esophagus, Diagnosis, Screening and surveillance

## Abstract

**Background:**

Esophageal adenocarcinoma (EAC) often presents at a late, incurable stage, and mortality has increased substantially, due to an increase in incidence of EAC arising out of Barrett’s esophagus. When diagnosed early, however, the combination of surgery and adjuvant therapies is associated with high cure rates. Metabolomics provides a means for non- invasive screening of early tumor-associated perturbations in cellular metabolism.

**Methods:**

Urine samples from patients with esophageal carcinoma (n = 44), Barrett’s esophagus (n = 31), and healthy controls (n = 75) were examined using ^1^H-NMR spectroscopy. Targeted profiling of spectra using Chenomx software permitted quantification of 66 distinct metabolites. Unsupervised (principal component analysis) and supervised (orthogonal partial least-squares discriminant analysis OPLS-DA) multivariate pattern recognition techniques were applied to discriminate between samples using SIMCA-P^+^ software. Model specificity was also confirmed through comparison with a pancreatic cancer cohort (n = 32).

**Results:**

Clear distinctions between esophageal cancer, Barrett’s esophagus and healthy controls were noted when OPLS-DA was applied. Model validity was confirmed using two established methods of internal validation, cross-validation and response permutation. Sensitivity and specificity of the multivariate OPLS-DA models were summarized using a receiver operating characteristic curve analysis and revealed excellent predictive power (area under the curve = 0.9810 and 0.9627 for esophageal cancer and Barrett’s esophagus, respectively). The metabolite expression profiles of esophageal cancer and pancreatic cancer were also clearly distinguishable with an area under the receiver operating characteristics curve (AUROC) = 0.8954.

**Conclusions:**

Urinary metabolomics identified discrete metabolic signatures that clearly distinguished both Barrett’s esophagus and esophageal cancer from controls. The metabolite expression profile of esophageal cancer was also discrete from its precursor lesion, Barrett’s esophagus. The cancer-specific nature of this profile was confirmed through comparison with pancreatic cancer. These preliminary results suggest that urinary metabolomics may have a future potential role in non-invasive screening in these conditions.

## Background

Once a rare malignancy, esophageal carcinoma is the sixth most frequent cause of death worldwide and the most rapidly rising cancer in the United States [1,2]. Esophageal adenocarcinoma (EAC) represents over 50% of all esophageal cancers in the western world, and the incidence of EAC arising from Barrett’s esophagus (BE) continues to increase at an alarming rate [1,3,4]. Nearly 60% of cases are diagnosed at an advanced, incurable stage; however, if diagnosed early these cancers are highly curable with a combination of surgical, endoscopic and adjuvant therapies [5]. Expected five-year survival rates for stage I disease approach 60% to 90%, highlighting the importance of developing effective screening tools to facilitate early diagnosis [4,6,7].

The majority, if not all cases of EAC arise from a region of BE, a well-recognized premalignant condition and common complication of gastro-esophageal reflux disease (GERD) affecting 12% to 20% of patients suffering from reflux [2,5,8,9]. BE remains the strongest individual risk factor for development of EAC and the only known precursor lesion [8]. Despite the presence of a well-defined histopathologic metaplasia-dysplasia-carcinoma sequence, current screening strategies for early detection have failed to reduce mortality from esophageal cancer in randomized prospective trials, and many studies have questioned their cost-effectiveness [9,10].

New sensitive tools are needed to overcome shortcomings of current screening and surveillance approaches for BE and esophageal cancer. Urinary metabolomics may offer one such potential for non-invasive screening of early tumor-associated perturbations in cellular metabolism. Identification of a discrete, urinary metabolomic signature associated with esophageal cancer and its precursor lesion, could allow for non-invasive screening in targeted, high-risk populations while helping to further elucidate underlying molecular pathogenesis and progression of disease. Furthermore, knowledge gained from metabolomics-based research could help advance personalized therapeutic approaches guided by early metabolic responses before phenotypic changes develop.

Currently, application of metabolomics-based techniques in the investigation of esophageal cancer remains limited. The majority of studies are serum-based and have included patients with late stage disease. The current case–control study sets out to compare the urinary metabolomic profiles of patients with early stage or locally advanced esophageal carcinoma and BE with healthy, age- and gender-matched controls. We hypothesize that global metabolite analysis of urine using NMR spectroscopy combined with statistical pattern recognition reductive techniques will reveal a characteristic metabolomic signature associated with esophageal carcinoma and its precursor lesion BE.

## Methods

### Study outline and sample collection

This study was approved by the Alberta Cancer Research Ethics Board and the Human Research Ethics Board of the University of Alberta. Written and informed consent was obtained from all participants prior to study enrollment. Random urine samples were collected preoperatively from patients with esophageal carcinoma (n = 44), BE (n = 31) as well as pancreatic cancer (n = 32) in the Edmonton region (results pertaining to pancreatic cancer cohort currently in press, Annals of Surgical Oncology 2012). Samples were collected prior to the initiation of chemoradiation therapy in patients with malignancy. For all cases, histologic findings were obtained and follow-up data were available to ensure accurate disease classification. Controls (n = 75) were healthy, age- and gender-matched male and female volunteers with no declared history of malignancy. Breastfeeding or pregnant women were excluded from study enrollment, as were patients with uncontrolled bacterial, viral or fungal infection. Additionally, subjects with compromised renal function reflected by impaired creatinine clearance were excluded to avoid confounding effects of impaired metabolite excretion. Patients with stage IV esophageal carcinoma were also excluded, as the aim of this study was the development of a metabolomic profile with potential uses in early detection and screening.

Urine samples were stored at −80°C prior to NMR analysis and within five hours of collection. Before data acquisition, samples were thawed and prepared by adding 75 μl of a chemical shift standard (Chenomx Inc., Edmonton, AB, Canada) containing 5.046 mM sodium 2,2-dimethyl-2-silapentane-5-sulfonate-d6 (DSS-d6) and 0.2% NaN3 in 99.8% w/v D20) to 675 μl of urine. The pH was adjusted using small additions of NaOH or HCl to obtain a final pH of 6.75 +/− 0.05 in order to reduce pH variation among samples. A 700 μl aliquot of prepared sample was then transferred to a 5mm NMR tube (Wilmad, Nuena, NJ, USA) immediately prior to NMR acquisition.

### ^1^H-NMR spectroscopic acquisition

^1^H-NMR spectra were acquired according to previously published and accepted methods [11,12]. Briefly, one-dimensional ^1^H-NMR spectra of urine samples were optimized, and excitation pulse calibrated based on single pulse nutation [13,14]. Spectra were acquired using the first increment of a standard nuclear Overhauser effect spectroscopy (NOESY) pulse sequence [15]. Experiments were executed on a two channel 600 MHz VNMRS spectrometer (Agilent Inc., Palo Alto, CA, USA) equipped with a 5mm-HX dual tune probe. Spectra were acquired at 25°C with an observation width of 12 PPM, 100-ms mixing time, 4 second acquisition time, 4 steady state scans, and 32 transients. Water suppression was achieved utilizing an 80 to 90 Hz gammaB1 1H continuous wave saturation pulse applied on the optimized water resonance during the 0.9s presaturation period and throughout the 100-ms mixing time. All spectra were zero-filled to 131k data points followed by apodization with a line-broadening weighting function of 0.5 Hz.

### Targeted profiling of spectra

Using Chenomx NMR Suite 7.0 software (Chenomx Inc.), metabolites were identified and quantified using a targeted profiling approach [16,17]. This method compares the integral of a known reference signal, DSS, with signals derived from a documented database of 297 compounds in order to determine concentrations relative to the reference signal [11]. All samples were analyzed blindly in a random fashion. A minimum of two analysts independently analyzed the spectra and only those compounds whose identity and concentrations were agreed upon were included. A set of 65 metabolites was identified and quantified. Additionally, creatinine concentrations of 12 randomly selected urine samples were verified using non-NMR, laboratory-based colorimetric techniques using a commercially available kit (Arbor Assays, DetectX Urinary Creatinine Kit, Cat K002-H5, Ann Arbour, Michigan, USA), and 95% correlation was achieved.

### Data analysis

Prior to further analysis, certain specific drug metabolites and drug vehicle constituents were excluded (ibuprofen, acetaminophen, salicylurate, proprionate, propylene glycol and mannitol). A number of metabolites were excluded due to low yield of detection among both cases and controls (gluconate, glycerol, ornithine, serine, 3-hydroxybutyrate, ethanol, uracil, adipate, and ascorbate). Signal overlap and poor signal shape make it more difficult to accurately detect (I do not accept this change) and quantify metabolites present in very low concentrations during the spectral deconvolution phase of analysis [16]. Therefore, those metabolites detected in less than 50% of cases and controls were excluded. The remaining 51 metabolites were included in all subsequent model developments. A complete list of all metabolites included in model development and their corresponding chemical shifts and multiplicities are available (see Additional file 1).

Metabolite concentrations were log-transformed to account for non-normal distribution of metabolite data, mean-centered to improve interpretability of the models generated and scaled to unit variance to ensure all metabolites, both high range and low range, were given equal weight in analysis. Creatinine normalization was attempted and made no significant difference to the predictive accuracies of all generated models. As such, raw, non-normalized data were used in concordance with several recent studies [12,18,19]. Patient characteristics were compared using Welch’s two-sample t-test for continuous variables and exact methods for categorical variables. Metabolite differences between groups were compared using Mann–Whitney non-parametric statistical analysis. Statistical significance was set at *P* <0.05. GraphPad Prism Version 5.0c was used for all descriptive statistics (GraphPad Software, San Diego, CA, USA).

Unsupervised (principal component analysis, PCA) and supervised (orthogonal partial least-squares discriminant analysis, OPLS-DA) multivariate pattern recognition techniques were applied to pre-processed metabolite concentration data to discriminate between sample spectra of cases and controls using SIMCA-P+ (version 12, Umetrics, Umeå, Sweden). By reducing the dimensionality of a set of measured variables, PCA provides a crude dataset overview and is used for initial exploratory analysis. For class discrimination, OPLS-DA with an integrated orthogonal signal correction (OSC) filter was applied. Partitioning of predictor variables improves both model transparency and interpretability [20,21].

Cross validation and permutation testing were applied for internal validation [22-24]. Predictive accuracy of the OPLS-DA models was summarized in terms of sensitivity and specificity using receiver operating characteristics (ROC) curves generated from cross-validated Y-predicted values (SIMCA-P+ software, Y-predcv, predictive Y). Area under the ROC curve (AUROC) was calculated using STATA/SE 10.1 (Stata, College Station, TX, USA). The variable importance on projection (VIP)-parameter was generated for a weighted, quantitative measure of discriminatory power of the metabolites; represented by a unitless number, the higher the value, the greater the discriminatory power of the metabolite. VIP scores >1 generally represent those metabolites carrying the most class discriminating information [24].

## Results

### Patient characteristics

Relevant patient and tumor characteristics are outlined in Table 1. There were no differences in age and gender when comparing patients with esophageal cancer (n = 44) and BE (n = 31) with healthy controls (n = 44, n = 31, respectively), as patients were matched based on these criteria. All cases of BE and esophageal cancer were confirmed histologically. The vast majority of cancers were EAC (72.7%). The majority of patients had stage I-IIIa disease (54.5%), involving the lower esophagus or esophagogastric junction. Sixty-one percent of cases had surgically resectable disease. A significant proportion of patients (68.2%) reported weight loss at the time of diagnosis (≥5% over 6 to 12 months). The majority of patients with BE had short segment disease (77.4%) and only one patient had low-grade dysplasia. The average time from initial diagnosis in this cohort of patients was 2.3 years. Demographic data associated with pancreatic cancer cases is published separately (in press, Annals of Surgical Oncology 2012).

**Table 1:** Clinical features of study subjects and tumor characteristics

**Number of subjects (n)**	**Esophageal Ca (44)**	**Controls (44)**	***p***	**Barrett's (31)**	**Controls (31)**	***p***
**Age** [median (range)]	63 (40-86)	62 (41-84)	0.824	64.5 (38-81)	64.5 (36-80)	1.000
**Gender** (male/female)	32/12	32/12	1.000	19/12	19/12	1.000
**TNM Stage**						
Ia/b	3/44 (6.8%)	**-**	**-**	**-**	**-**	**-**
IIa/b	11/44 (25%)	**-**	**-**	**-**	**-**	**-**
IIIa	10/44 (22.7%)	**-**	**-**	**-**	**-**	**-**
IIIb	6/44 (13.6%)					
IIIc	6/44 (13.6%)					
Unknown	8/44 (18.2%)	**-**	**-**	**-**	**-**	**-**
**Histologic Type**			**-**			**-**
Esophageal Adenocarcinoma	32/44 (72.7%)	**-**	**-**	**-**	**-**	**-**
Squamous Cell Carcinoma	11/44 (25%)	**-**	**-**	**-**	**-**	**-**
Poorly Differentiated	11/44 (2.3%)					
**Histologic Grade**			**-**			**-**
1	4/44 (9.1%)	**-**	**-**	**-**	**-**	**-**
2	17/44 (38.6%)	**-**	**-**	**-**	**-**	**-**
3	17/44 (38.6%)	**-**	**-**	**-**	**-**	**-**
Unavailable	6/44 (13.6%)	**-**	**-**	**-**	**-**	**-**
**Location**			**-**			**-**
Cervical	1/44 (2.3%)	**-**	**-**	**-**	**-**	**-**
Upper Thoracic	1/44 (2.3%)	**-**	**-**	**-**	**-**	**-**
Middle Thoracic	8/44 (18.2%)					
Lower Thoracic/EGJ	35/44 (79.5%)					
**Resectable**	27/44 (61.4%)	**-**	**-**	**-**	**-**	**-**
**Unresectable**	17/44 (38.6%)	**-**	**-**	**-**	**-**	**-**
**Barrett's Esophagus**						
Short segment (<3cm)				24/31 (77.4%)		
Long segment (≥3cm)				8/31 (25.8%)		
Dysplasia						
Low grade				1/31 (3.2%)		
High grade				0/31 (0%)		
Average time from initial diagnosis (yr)				2.3		
**Weight Loss (≥5%)**			**-**			**-**
Yes	30/44 (68.2%)	**-**	**-**	**-**	**-**	**-**
No	6/44 (13.6%)	**-**	**-**	**-**	**-**	**-**
Unavailable	8/44 (18.2%)	**-**	**-**	**-**	**-**	**-**

Ca, carcinoma.

### Metabolomic profile associated with esophageal carcinoma

**Figure 1:**
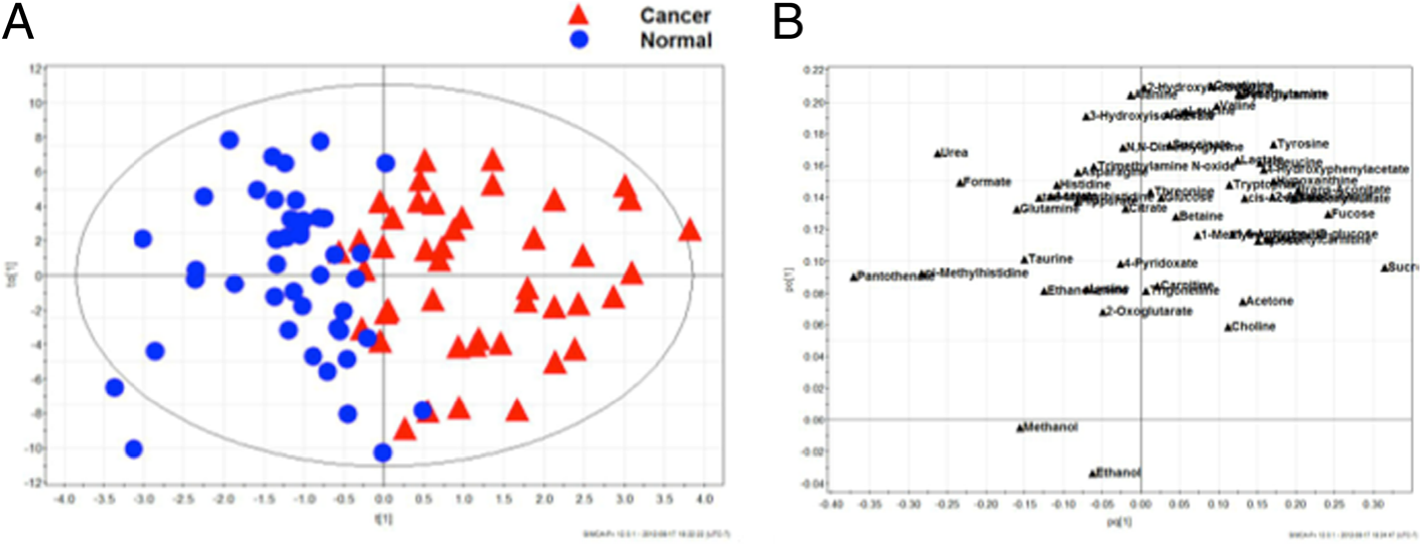
**OPLS-DA score plot of metabolite profiles derived from esophageal carcinoma and healthy controls with corresponding loading plot.A**) Supervised OPLS-DA score plot. Two-component model based on 53 measured urinary metabolites. Esophageal cancers are represented by red triangles and blue circles depict controls. **B**) OPLS-DA loading plot of urinary metabolite profiles derived from esophageal carcinoma and healthy controls. OPLS-DA, orthogonal partial least squares discriminant analysis.

**Figure 2:**
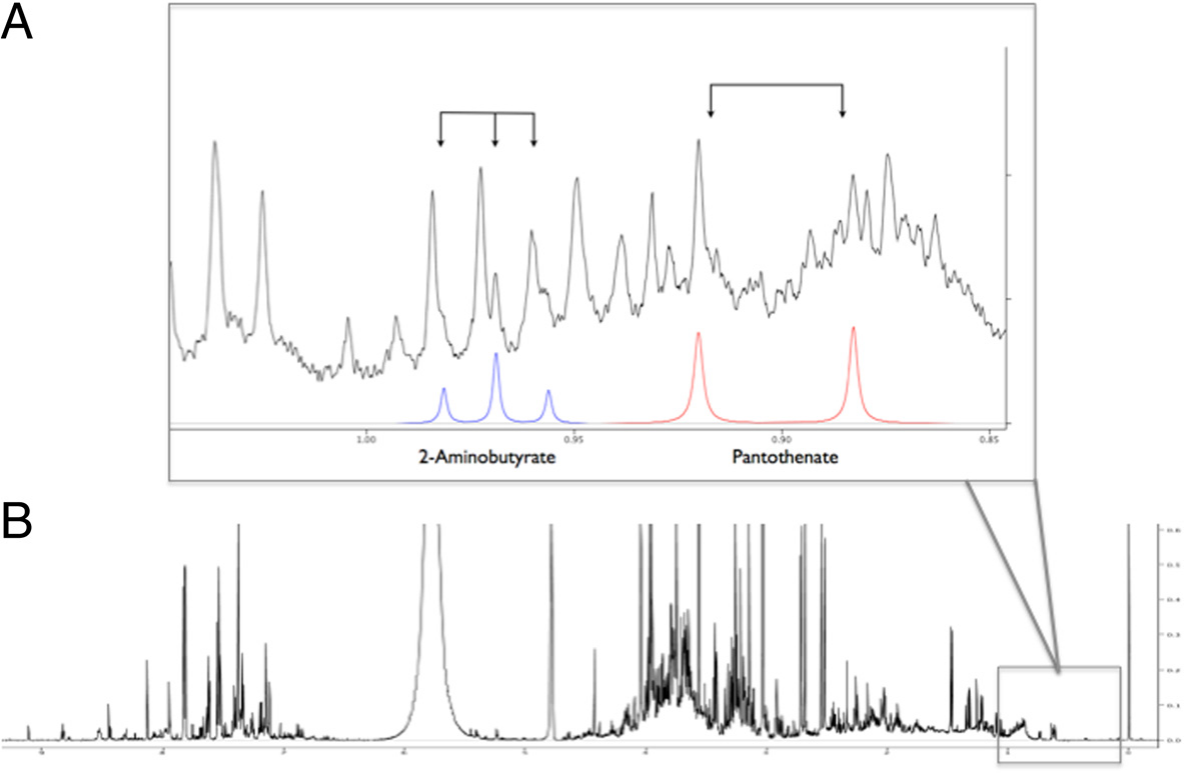
**Representative urine **^**1**^**H-NMR spectrum of esophageal cancer.** Urinary ^1^H-NMR spectrum of esophageal cancer. X-axis = chemical shift relative to internal standard, DSS-d6, Y-axis = peak amplitude relative to DSS-d6. **A**) Full ^1^H-NMR spectrum. **B**) Representative region of spectrum with labeled metabolites. Black line represents ^1^H-NMR spectrum, blue line represents compound signature corresponding to 2-aminobutyrate, red line represents compound signature corresponding to pantothenate. DSS-d6, sodium 2,2-dimethyl-2-silapentane-5-sulfonate-d6.

Metabolite concentration data of stage I-III esophageal cancer patients were analyzed using both unsupervised (PCA) and supervised (OPLS-DA) multivariate pattern recognition methods. Even at the exploratory, unsupervised phase of analysis, suggestion of group clustering based on disease status (cancer versus healthy) was observed (see Additional file 2). Supervised pattern recognition techniques were then applied using OPLS-DA to compare the metabolite expression profiles of patients with esophageal cancer and healthy controls. The outcome demonstrated clear separation of patients with cancer from controls (Figure 1A). Corresponding loading plots for the above (Figure 1B) and all subsequent models are also available (see Additional files 3, 4, and 5). Furthermore, a sample ^1^H-NMR spectrum of esophageal cancer demonstrating several characteristic metabolites is also demonstrated (Figure 2).

**Figure 3:**
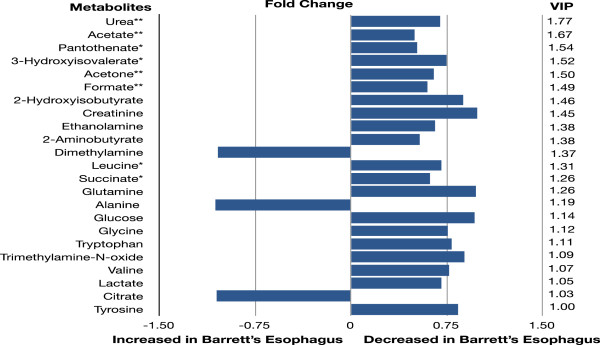
Key metabolite differences between esophageal cancer and healthy controls.

Mann–Whitney statistical analysis was used to compare the individual metabolite concentrations between patients with esophageal cancers and controls. Nine metabolites had significantly different levels of expression (*P* value range for nine significant metabolites = *P* <0.0001 to 0.0426). Metabolites exhibiting significant concentration differences and those contributing the most class discriminating information based on the VIP are listed in Figure 3, together with the results of fold change calculations. It is apparent that simultaneous perturbations in multiple metabolic pathways contribute to the observed class separation.

Key metabolites involved in OPLS-DA model according to VIP- parameter and *P*-value significance. Only those metabolites with significant concentration differences or a VIP-parameter ≥1 are displayed. *P*-values were obtained using Mann–Whitney nonparametric statistical analysis. *P <0.05, **P <0.01, ***P <0.0001. Fold change was calculated by dividing the median metabolite concentration in cancers by controls.

A sub-analysis was also carried out excluding exogenous metabolites in order to minimize dietary or environmental influences on model generation. These included sucrose, 1,6-anhydro-D-beta-glucose, adipate, 2-hyroxyisobutyrate, ascorbate, ethanol and xylose. Methanol (a microbial metabolite) was also excluded in the secondary analysis. A more detailed description of these compounds is also available (see Additional file 6). The OPLS-DA model achieved comparable class separation, with similar predictive accuracy (AUROC = 0.9619, results not shown). These results suggest that exogenous metabolites did not contribute significantly to class discrimination. The identity and sequence of key discriminating metabolites based on VIP-analysis was unaltered following the exclusion of the exogenous metabolites with the exception of the inclusion of 1-methylnicotinamide (precursor molecule to coenzymes nicotinamide-adenine dinucleotide (NAD+) and nicotinamdie-adenine dinucleotide phosphate (NADP+), metabolites involved in energy metabolism), 4-hydroxyphenylacetate (amino acid derivative) and isoleucine (amino acid). These metabolites were not predominant, however, with VIP-values very close or equal to 1.0.

### Metabolomic profile associated with Barrett’s esophagus

**Figure 4:**
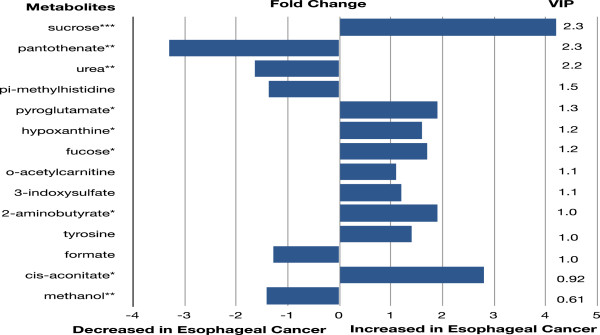
Key metabolite differences between Barrett’s esophagus and healthy controls.

Metabolite concentration data of patients with BE and healthy controls were also compared using multivariate pattern recognition techniques in order to determine if the metabolite expression profile of this pre-malignant lesion was distinct from that of healthy controls. While group clustering based on disease status was not observed at the unsupervised phase of analysis, clear class separation was achieved when supervised methods were applied (see Additional file 7). These results suggest the presence of detectable metabolic disturbances even at the pre-invasive stage of esophageal cancer progression. Eight metabolites were differentially expressed when comparing patients with BE to healthy controls using Mann Whitney statistical analysis (*P* value range for eight significant metabolites = *P* <0.05 to 0.01). Key metabolites most responsible for class separation based on the VIP-parameter and significant concentration differences are listed in Figure 4, together with their respective fold change.

Key metabolites involved in the OPLS-DA model according to VIP-parameter and *P*-value significance. Only those metabolites with significant concentration differences or a VIP-parameter ≥1 are displayed. *P*-values were obtained using Mann–Whitney nonparametric statistical analysis. Fold change was calculated by dividing the median metabolite concentration in BE by controls.

Finally, concentration data of patients with early stage/locally advanced esophageal cancer and BE were compared using similar methods of analysis. In this model, esophageal cancer patients with squamous cell carcinoma on histology were excluded. While group clustering was not achieved at the unsupervised phase of analysis when supervised methods were applied, class separation became clearly apparent when comparing the metabolite expression profiles of patients with EAC and BE (see Additional file 8). As anticipated, a degree of overlap not present when comparing esophageal cancer patients to healthy controls was evident. This overlap was entirely expected based on the known and histologically proven background BE in these patients developing EAC.

Unique patterns of metabolite expression were observed when comparing metabolite concentration data from patients with EAC and BE. Mann Whitney analysis revealed 10 metabolites with significant concentration differences (*P* value range for 10 significant metabolites = *P* <0.043 to 0.0003). As expected, while there were similarities in the metabolite expression patterns when comparing these results to the model discriminating esophageal carcinoma from healthy controls, a number of unique metabolite differences were observed.

### Model validation and prediction accuracy

**Figure 5:**
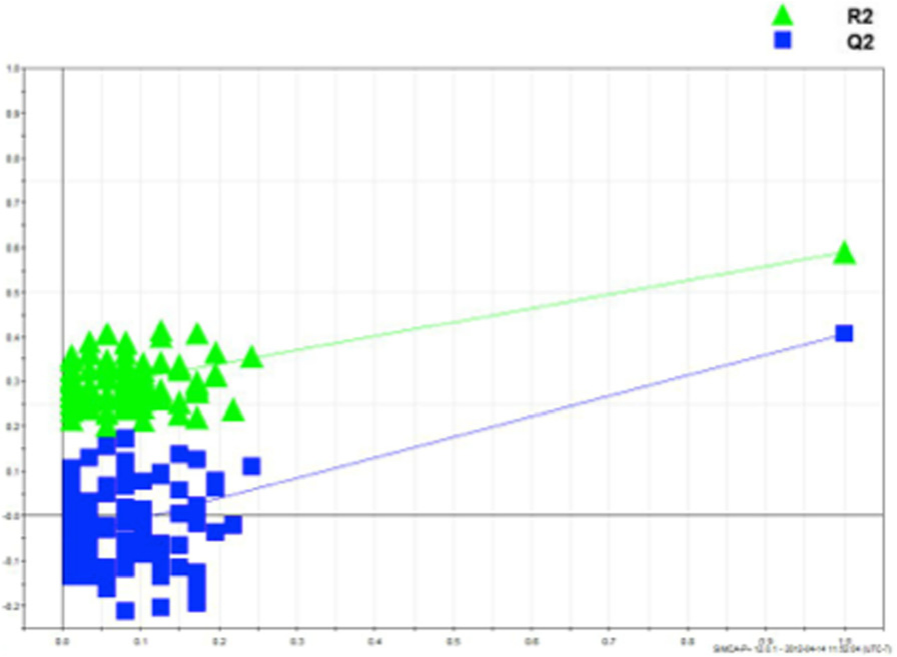
**Permutation analysis of OPLS-DA model derived from esophageal carcinoma versus healthy controls.** Statistical validation of the OPLS-DA model by permutation analysis using 100 different model permutations. The goodness of fit (R^2^) and predictive capability (Q^2^) of the original model are indicated on the far right and remain higher than those of the 100 permuted models to the left. OPLS-DA, orthogonal partial least squares discriminant analysis.

Permutation testing and cross validation, two established methods of internal validation, were used to confirm model validity. Permutation tests involve the random assignment of class labels to cases and controls. Permutation testing using 100 random permutations demonstrates that the goodness of fit and predictive ability (R^2^/Q^2^) of the original models discriminating esophageal cancers (Figure 5) and BE from controls was higher than those of the permuted models. This was also confirmed in the model comparing EAC to BE.

**Figure 6:**
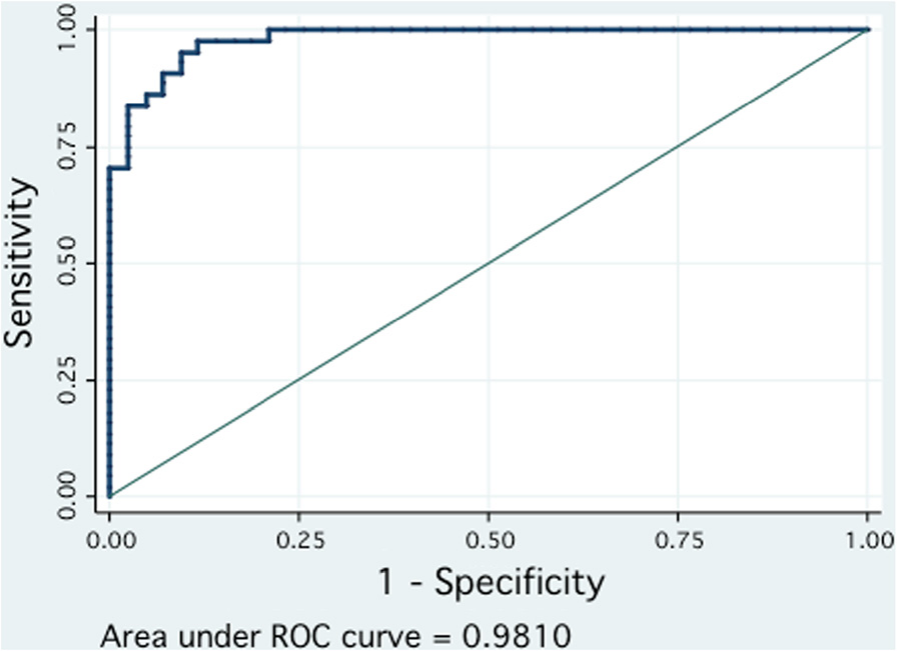
**Predictive accuracy of the OPLS-DA model discriminating esophageal carcinoma and healthy controls summarized using ROC curve analysis.** The ROC curve of the OPLS-DA model discriminating esophageal cancers and controls generated using cross-validated predicted-Y values of the OPLS-DA model. AUROC = 0.9810. Statistical validation of the OPLS-DA model by permutation analysis using 100 different model permutations. The goodness of fit (R^2^) and predictive capability (Q^2^) of the original model are indicated on the far right and remain higher than those of the 100 permuted models to the left. AUROC, area under the receiver operating curve; OPLS-DA, orthogonal partial least squares discriminant analysis; ROC, receiver operating curve.

OPLS-DA model generation employed a seven-fold cross validation step. This involves omitting a portion of the data from model development, developing parallel models from the reduced data, predicting the omitted data from the different models, and then comparing predicted with actual values, providing an estimate of overall predictive power. Using cross-validated Y-predicted values, model sensitivity and specificity were summarized using ROC curves for the models distinguishing esophageal cancer (AUROC = 0.9810) and BE (AUROC = 0.9627) from healthy controls (Figure 6 and Additional file 7, respectively). Results were indicative of strong predictive power. Predictive accuracy of the model distinguishing EAC from BE was less pronounced, as expected, with an AUROC = 0.9430 (see Additional file 8).

#### Cancer-specific metabolomic profile

In order to determine whether the patterns of altered metabolite expression observed in esophageal carcinoma were reflective of metabolic changes common to all malignancies or whether these disturbances were specific to esophageal carcinoma, we compared the metabolomic profiles of two disparate malignancies. When comparing metabolite concentration data of patients with esophageal cancer (n = 44) and pancreatic cancer (n = 32) using supervised methods (OPLS-DA), class separation was evident, indicating that the metabolic perturbations observed were likely reflective of metabolic alterations specific to esophageal cancer while areas of overlap were likely representative of shared common final pathways of tumorigenesis (see Additional file 9, AUROC = 0.8954).

## Discussion

Esophageal cancer is an aggressive malignancy with poor prognosis, in part due to delayed diagnosis. Nearly 50% of patients do not present until they are at an advanced, incurable stage; however, current surgical and adjuvant therapies offer high cure rates in early stage disease [5]. For most of the twentieth century, squamous cell carcinoma accounted for the vast majority of esophageal cancers. In the past three decades, however, the overall incidence of EAC has increased at an alarming rate, and this has been accompanied by dramatic increases in mortality [5]. While reasons for this steady progression in incidence remain largely unknown, they are thought to be secondary to increased rates of obesity and GERD. BE, affecting up to 20% of patients with GERD, is a well-recognized precursor lesion of EAC and carries a 30- to 125-fold increased risk of cancer development when compared with the general population [25].

Despite the presence of a known, stepwise, metaplasia-dysplasia-carcinoma sequence of cancer progression providing the opportunity for early detection, screening and treatment, there is little evidence that current surveillance strategies have prevented deaths resulting from EAC [25]. Traditionally, screening and surveillance strategies for EAC among patients with BE involve endoscopic detection with histopathologic confirmation of Barrett's metaplasia-dysplasia. Challenges impeding the effectiveness of current strategies aimed at early detection include the large number of patients with silent reflux harboring BE, the discontinuous nature of metaplastic and dysplastic epithelium when using a technique of random endoscopic biopsy and the absence of a well-validated risk stratification model capable of accurately and reliably identifying those individuals truly at risk of progression along the metaplasia-carcinoma sequence [26]. Moreover, there exists a subset of patients who progress to high-grade dysplasia and cancer with no evidence of dysplasia on previous recent surveillance endoscopies, representing a cohort of patients who may, therefore, evade detection using current screening strategies [26,27].

Urinary metabolomics offers a novel and sensitive approach to simultaneously evaluating tumor-associated perturbations of multiple metabolic pathways and their downstream functional significance prior to the surfacing of gross phenotypic change. Application of metabolomic tools could therefore provide an opportunity for screening and early detection, molecularly-guided and personalized therapeutics as well as further interrogation of the molecular pathogenesis of esophageal cancer progression.

There remains a dearth of research examining the role of metabolomics as a potential diagnostic or screening tool in esophageal cancer and its precursor lesion, BE. The majority of studies have been serum- or tissue-based, limited in sample size and inclusive of patients with metastatic disease. As a biofluid, urine has a number of distinct advantages, namely related to ease of sample collection, storage, processing and reproducibility. Furthermore, urinary metabolomics avoids the issue of degraded spectral resolution resulting from high lipid and protein content, often a complicating feature in the analysis phase of serum studies.

Djukoivc *et al*. used a targeted metabolite approach to investigate the potential role of nucleosides as cancer biomarkers in EAC [28]. They uncovered a number of differences in nucleoside expression when comparing cancer patients to healthy controls. Using a serum-NMR based approach, Zhang *et al*. were able to discriminate patients with EAC from healthy controls, in congruence with results of other serum studies of smaller sample size [29-31]. They also found that patients with BE and high-grade dysplasia appeared to overlap with both cancers and controls; however, this analysis was limited to five patients. Moreover, patients with stage IV disease were included and, while a subgroup analysis was done, potential confounding effects of age and gender were not taken into account at the primary stage of analysis.

Using a representative sample of patients with early stage or locally advanced disease, we have identified a discrete and specific urinary metabolomic signature of esophageal cancer which is clearly distinguishable from healthy controls and exhibits strong predictive accuracy. Moreover, even at the pre-invasive stage of disease, metabolomic profiling accurately distinguishes patients with BE. Esophageal cancer is a highly curable malignancy when treated in its early stages. A non-invasive, highly predictive screening tool capable of early disease detection could, therefore, have significant impact on the management of esophageal cancer with the potential to drastically alter outcomes.

Biochemical interpretation of the altered patterns of metabolite expression when comparing esophageal cancers with controls must be made with caution since it is likely that the overall metabolite expression profile results from a convergence of changes occurring at the tumor microenvironment level and disturbances in overall global metabolism. We suspect that cancer-specific elevations of 2-aminobutyrate (a key intermediate in the biosynthesis of opthalmate which is a tripeptide analogue of glutathione) may be reflective of increased oxidative stress [32,33]. Cancer cells both *in vitro* and *in vivo* are thought to be under continuous oxidative stress, in part resulting from outgrowth of blood supply occurring with tumor proliferation [34]. Esophageal cancers grow and spread rapidly, with lymph node metastases in nearly 20% of T1 lesions and 60% of T2 lesions [5]. Likewise, cancer-specific elevations of hypoxanthine may represent an underlying enhanced capacity for DNA synthesis in association with tumor growth. Our findings of cancer-specific decreases of pantothenate, a precursor of coenzyme A and elevations of cis-aconitate, a tricarboxylic acid cycle intermediate, were in agreement with observations of Ikeda *et al*. and may be a result of aggressive tumor growth with resulting increased energy demands [29]. It is possible that elevated levels of fucose among cancer patients may be associated with subclinical hepatocellular injury from early metastatic disease, as this metabolite is often secreted in association with liver damage [33].

Cachexia is present in up to 80% of patients with malignancies of the upper gastrointestinal tract and is a complicating feature in a significant proportion of patients with esophageal cancer [35]. Significant elevations of pi-methylhistidine were observed among cancer patients and may be reflective of cachexia-induced skeletal muscle protein breakdown. Trends towards significance were observed with regard to cancer-specific elevation of a number of amino acid and amino acid derivatives (tyrosine, tryptophan, threonine, trigonelline, 4-hydroxyphenylacetate and isoleucine), possibly resulting from muscle wasting in these patients. In the early stages of malignancy, cachexia-associated muscle wasting is often an occult phenomenon [12,36]. A noninvasive means of detecting early muscle wasting in patients harboring occult or early malignancy could have significant clinical utility.

As expected, there was a larger degree of overlap when comparing the metabolite expression profiles of patients with EAC and BE compared to those of healthy controls. Barrett’s metaplasia was noted in over 50% of final surgical specimens resected in the setting of EAC. Accordingly, several key metabolites were common to the models discriminating esophageal cancers from healthy controls and BE. Metabolite differences observed when comparing patients with BE to healthy controls may in part be reflective of the hyperproliferative state known to characterize Barrett’s epithelium [1,26]. Relative decreases in the concentration in a number of metabolites among patients with BE (succinate, pantothenate, acetate, formate) may be reflective of this hyperproliferative state and a resultant overall increased cellular energy demand. Additionally, preferential use of branch chain amino acids, specifically leucine, is characteristic of stress states. Lower levels seen among patients with BE may have been reflective of the metabolic stress associated with low grade, chronic inflammation [33]. At this stage, however these postulations remain speculative and further experimental *in vivo* modeling is required to confirm their relevance. Of note, disease chronicity, extent and severity appeared to be well correlated with degree of metabolic derangement. The metabolic profiles of those patients with a longstanding history of BE (mean four years), those with long segment BE or those with low grade dysplasia (one patient) showed the greatest degree of discrimination from that of normal controls.

In an effort to explore whether the metabolic disturbances observed were, in fact, specific to esophageal cancer or reflective of common final pathways of global metabolic change associated with malignancy, the metabolomic profiles of patients with esophageal and pancreatic cancer were compared. Key discriminatory metabolites identified through VIP-analysis revealed several distinguishing patterns of metabolic expression. Acetone (an end-product of ketogenesis) and glucose, two key discriminatory metabolites elevated among patients with pancreatic cancer, are perhaps reflective of underlying diabetogenic disturbances associated with this malignancy. Hyperinsulinemia and peripheral insulin resistance are metabolic perturbations frequently observed in pancreatic cancer [37,38]. Elevated levels of 3-indoxylsulfate, another key discriminatory metabolite, were observed among patients with esophageal cancer. This may perhaps be reflective of the prominent role of oxidative damage in the malignant transformation of esophageal cancer as 3-indoxylsulfate acts to propagate oxidative stress by strongly decreasing circulating levels of glutathione, a key cellular antioxidant [39,40].

While the metabolite expression profiles of patients with esophageal and pancreatic cancer were clearly distinguishable, a region of overlap was evident. This area of overlap could be reflective of metabolic changes associated with shared common pathways of tumorigenesis including increased energy expenditure associated with tumor cell proliferation and growth, other molecular commonalities involved in tumor angiogenesis, invasion and distant spread as well as cachexia-associated muscle wasting. The metabolites most contributory to the overlapping region were related to energy metabolism and cellular proliferation (1-methylhistidine, o-acetylcarnitine) as well as those reflective of muscle wasting, including pi-methylhistidine, creatinine, and a number of amino acids and amino acid derivatives (leucine, tyrosine, isoleucine, valine, tryptophan and 4-hydroxyphenylacetate).

While these preliminary results are encouraging, a number of limitations must be acknowledged including small sample size. Future studies will include an external validation step to further confirm model validity using an independent cohort of patients not involved in model development. The predictive accuracy of reduced models containing only those key discriminatory metabolites identified here (metabolites with significant concentration differences or a VIP-parameter ≥1) will also be tested at this stage.

While the potential confounding effects of chemoradiation treatments were controlled for by collecting samples prior to any therapeutic intervention, one limitation of this study could be the potential confounding effect of medication. It was impossible, however, in a study of this size and design to control for this potential confounder given the wide range of medications being prescribed among enrolled patients. We suspect, however, that any effect was likely small and not relevant as considerable differences in the medication profiles were not apparent on initial analysis. Furthermore, while drug metabolites and drug vehicle constituents were excluded from analysis, it was not possible for us to control for the potential downstream effects of these medications on the expression of other metabolites. While this could be a potential confounder, we believe the effect is small and likely reasonably balanced between groups.

An additional limitation of this study could have resulted from a lack of control for dietary factors. However, when a number of exogenous metabolites were excluded from analysis, predictive accuracy was maintained. While dietary factors were not directly controlled for, we have no clear reason to believe there would be any major systematic dietary differences between these patients given that a large proportion of the patients with esophageal cancers were early stage and the patients with BE were otherwise medically fit. Furthermore, given that all cases and all controls were not consuming identical diets, it is more likely that any dietary effects were responsible for creating background noise, potentially obscuring further important, biological patterns. This issue could be addressed further in future studies, however, through the use of dietary records to account for any gross dietary differences.

Future studies should also integrate results from both serum and urine analysis, while using diverse analytical platforms such as gas-chromatography or liquid chromatography-mass spectrometry in order to gain a more complete assessment of the metabolic milieu associated with esophageal cancer and its precursor lesion, BE.

## Conclusions

Using NMR and multivariate statistical techniques, we identified a discrete urinary metabolic signature associated with early and locally advanced esophageal cancer. The metabolic profile of esophageal cancer was distinct from that of healthy controls and demonstrated strong predictive power. We also identified a highly accurate, metabolic signature of the precursor lesion, BE. Identification of a highly sensitive and specific non-invasive tool capable of early disease detection could allow for improved population-based screening of high-risk populations, allowing for early intervention at the pre-invasive or early stage of disease when cure rates are high. Furthermore, greater understanding of the molecular pathogenesis of BE and esophageal cancer carcinogenesis could reveal new molecular targets to further optimize and personalize current adjuvant therapies.

## Abbreviations

AUROC: Area under the receiver operating characteristics curve; BE: Barrett’s esophagus; DSS-d6: Sodium 2,2-dimethyl-2-silapentane-5-sulfonate-d6; EAC: Esophageal adenocarcinoma; GERD: Gastroesophageal reflux disease; NMR: Nuclear magnetic resonance; NOSEY: Nuclear Overhauser effect spectroscopy; OPLS-DA: Orthogonal partial-least squares discriminant analysis; OSC: Orthogonal signal correction; PCA: Principal component analysis; ROC: Receiver operating characteristics curve; VIP: Variable importance on projection.

## Competing interests

There are no competing interests to declare.

## Authors' contributions

VWD participated in study design, carried out sample processing and spectroscopy, completed statistical analysis and drafted the manuscript. DES was involved in study design and coordination, and helped to draft the manuscript. DE participated in study design and helped to draft the manuscript. MBS conceived the study, participated in its design and coordination and helped to draft the manuscript. All authors read and approved the final manuscript.

## Authors' information

MBS is an associate professor and medical oncologist at the University of Alberta with a broad background in metabolomics research, and has examined the role of metabolomics-based applications in the management of a number of other malignancies including ovarian and breast cancer as well as pancreatic cancer.

## Supplementary Material

Additional file 1 ^**1**^**H-Chemical Shift (ppm relative to DSS-d6) and Corresponding Multiplicities For All Identified Metabolites.** Click here for file

Additional file 2 **PCA Score Plot of Urinary Metabolite Profiles Derived from Esophageal Carcinoma and Healthy Controls.** Esophageal cancer samples are represented by red triangles and blue circles depict controls. Two-component model based on 53 measured metabolites. Click here for file

Additional file 3 OPLS-DA Loading Plot OPLS-DA of Metabolite Profiles Derived From BE and Healthy Controls. Click here for file

Additional file 4 OPLS-DA Loading Plot of Metabolite Profiles Derived From EAC and BE. Click here for file

Additional file 5 OPLS-DA Loading Plot of Metabolite Profiles Derived From Esophageal and Pancreatic Cancer. Click here for file

Additional file 6 Exogenous Metabolites with Source Description. Click here for file

Additional file 7 **OPLS-DA Score Plot of Metabolite Profiles Derived from BE and Healthy Controls with Corresponding ROC Curve Analysis. A)** Supervised OPLS-DA score plot. Two-component model based on 53 measured urinary metabolites. BE is represented by red triangles and controls are depicted by blue circles**. B)** Corresponding ROC curve generated using cross-validated predicted-Y values of OPLS-DA model. AUROC = 0.9627. Click here for file

Additional file 8 **OPLS-DA Score Plot of Metabolite Profiles Derived from EAC and BE with Corresponding ROC Curve Analysis. A)** Supervised OPLS-DA score plot. Two-component model based on 53 measured urinary metabolites. BE is represented by blue circles triangles and EAC is depicted by red triangles**. B)** Corresponding ROC curve generated using cross-validated predicted-Y values of OPLS-DA model. AUROC = 0.9430. Click here for file

Additional file 9 **OPLS-DA Score Plot Depicting Cancer Specificity of Urinary Metabolomic Profiles and Corresponding ROC Curve Analysis. A)** Urinary metabolomic profiles of patients with esophageal carcinoma represented by blue squares, and pancreatic ductal adenocarcinoma, depicted by red triangles. **B)** Corresponding ROC curve generated using cross-validated predicted-Y values of OPLS-DA model. AUROC = 0.8954. Click here for file
