# Supplementary material for: Urinary metabolomic signature of esophageal cancer and Barrett’s esophagus
Source: World J Surg Oncol. 2012 Dec 15;10:271. doi: 10.1186/1477-7819-10-271 (PMC3579706; doi:10.1186/1477-7819-10-271)
Supplement: Additional file 1 — 1H-Chemical Shift (ppm relative to DSS-d6) and Corresponding Multiplicities For All Identified Metabolites. [file 1477-7819-10-271-S1.pdf]

Metabolite	^{1}H chemical shift (ppm relative to DSS) and multiplicity[a]
1-Methylnicotinamide	9.26(br s), 8.95(m), 8.88(m), 4.47(s)
1,6-Anhydro-β-D Glucose	5.44(m), 4.98(dd)
2-Aminobutyrate	0.97(t)
2-Hydroxyisobutyrate	1.35(s)
2-Oxoglutarate	3.00(t), 3.00(t)
3-Hydroxyisovalerate	2.36(s), 1.26(s)
3-Indoxysulfate	7.69(m), 7.49(m), 7.26(m), 7.19(m)
4-Hydroxyphenylacetate	7.15(m), 6.85(m), 3.44(s)
4-Pyridoxate	7.86(s), 2.44(s)
Acetate	1.91(s)
Acetone	2.22(s)
Alanine	1.47(d)
Asparagine	2.95(dd), 2.85(dd)
Betaine	3.25(br s)
Carnitine	3.21(br s), 2.43(br s)
Choline	3.19(br s)
cis-Aconitate	3.12(d), 5.74(t)
Citrate	2.53(d), 2.68(d)
Creatinine	3.03(br s), 4.05(br s)
Dimethylamine	2.72(s)
Ethanolamine	3.14(m)
Ethylmalonate	1.70(m), 0.87(t)
Formate	8.44(s)
Fucose	5.20(d), 4.55(d), 1.24(d)
Glucose	5.23(d), 4.63(d), 3.23(m)
Glutamine	2.43(m), 2.46(m)
Glycine	3.55(s)
Hippurate	7.82(m), 7.63(M), 7.54(m), 3.96(d)
Histidine	7.85(br s), 7.08(br s)
Hypoxanthine	8.20(s), 8,18(s)
Isoleucine	1.00(d), 0.93(t)
Lactate	1.32(d)
Leucine	0.95(d), 0.94(d)
Lysine	3.02(m), 1.71(m)
N,N-Dimethylglycine	2.91(s)
O-Acetylcarnitine	3.18(br s), 2.13(s)
Pantothenate	0.92(s), 0.88(s)
π-Methylhistidine	8.07(br s), 7.12(br s), 3.73(s)
Pyroglutamate	2.39(m)
Succinate	2.40(s)
Sucrose	5.40(d), 4.21(d)
τ-methylhistidine	7.72(br s), 7.02(br s)
Taurine	3.42(t), 3.25(t)
Threonine	1.32(d)
trans-Aconitate	6.58(t), 3.44(d)
Trigonelline	9.11(br s), 8.82(m),4.42(s)
Trimethylamine-N-oxide	3.25(s)
Tryptophan	7.71(m)
Tyrosine	7.18(m), 6.88(m)
Urea	5.77(br s)
Valine	1.03(d), 0.98(d)
Xylose	4.57(d)

[a] *s* (singlet), *br s* (broad singlet), *d* (doublet), *dd* (doublet of doublets), *t* (triplet)
