# Supplementary figures and images for: Urinary metabolomic signature of esophageal cancer and Barrett’s esophagus
Source: World J Surg Oncol. 2012 Dec 15;10:271. doi: 10.1186/1477-7819-10-271 (PMC3579706; doi:10.1186/1477-7819-10-271)

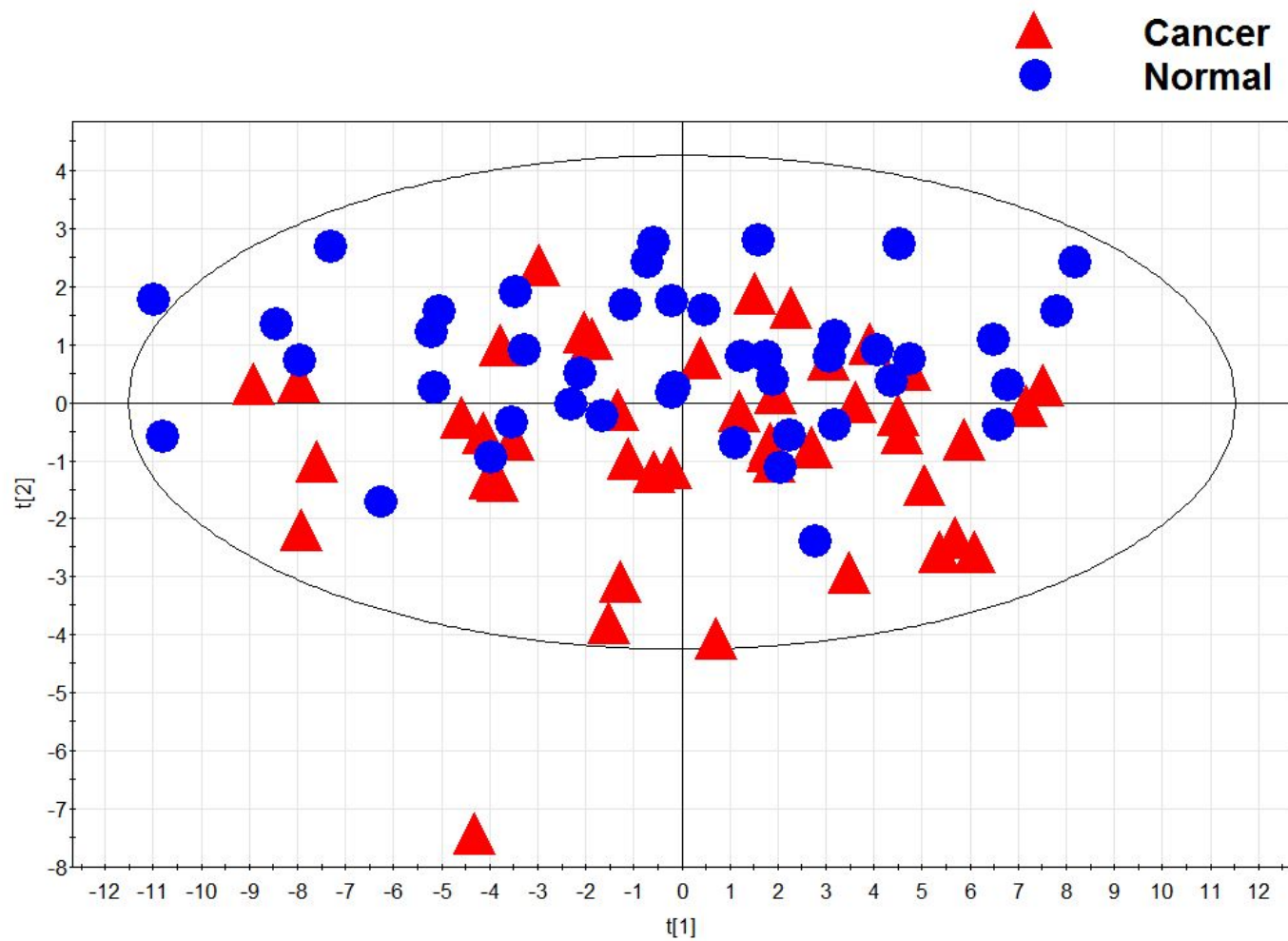

Supplement: Additional file 2 — PCA Score Plot of Urinary Metabolite Profiles Derived from Esophageal Carcinoma and Healthy Controls. Esophageal cancer samples are represented by red triangles and blue circles depict controls. Two-component model based on 53 measured metabolites. [file 1477-7819-10-271-S2.pdf]

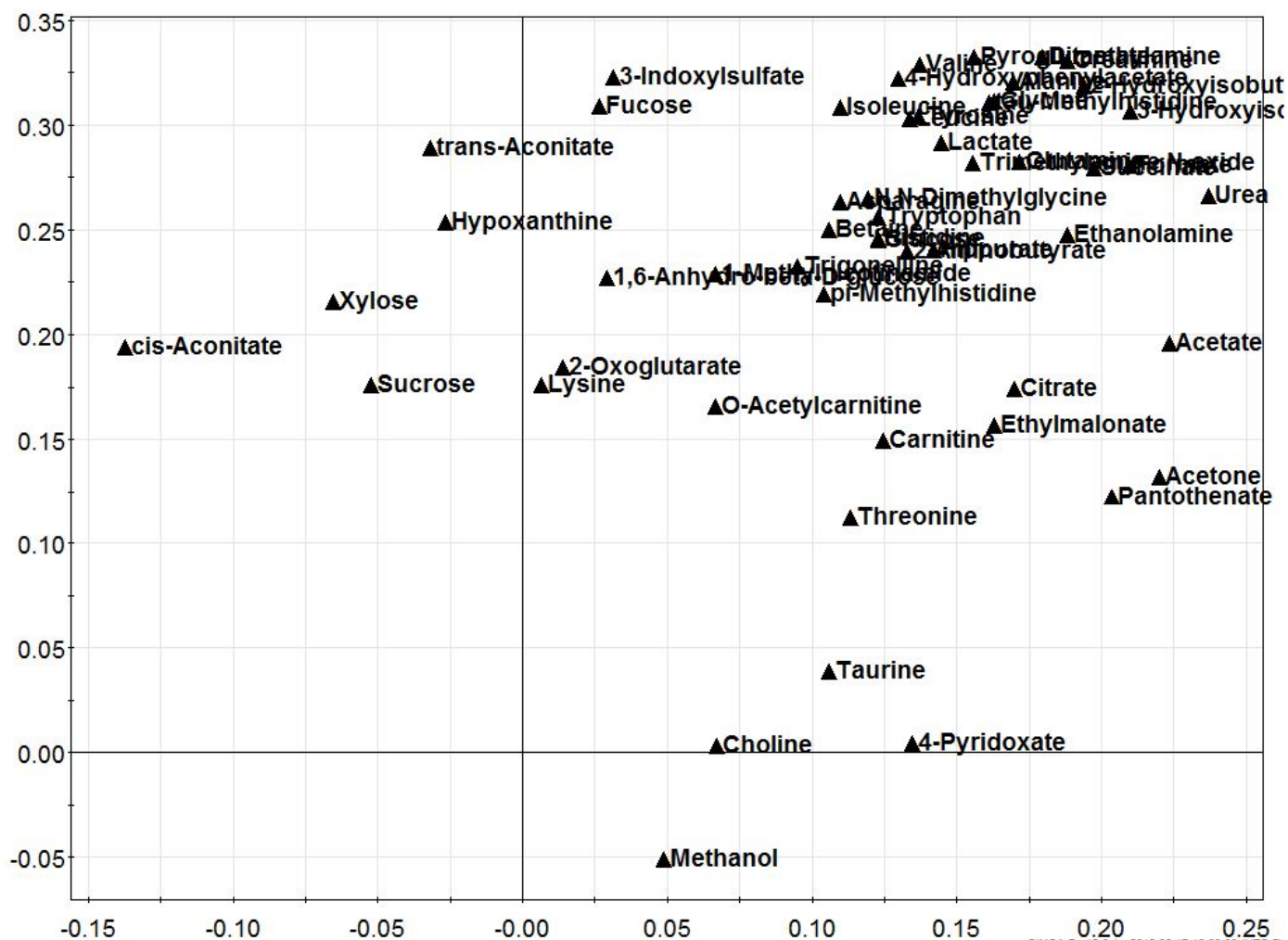

Supplement: Additional file 3 — OPLS-DA Loading Plot OPLS-DA of Metabolite Profiles Derived From BE and Healthy Controls. [file 1477-7819-10-271-S3.pdf]

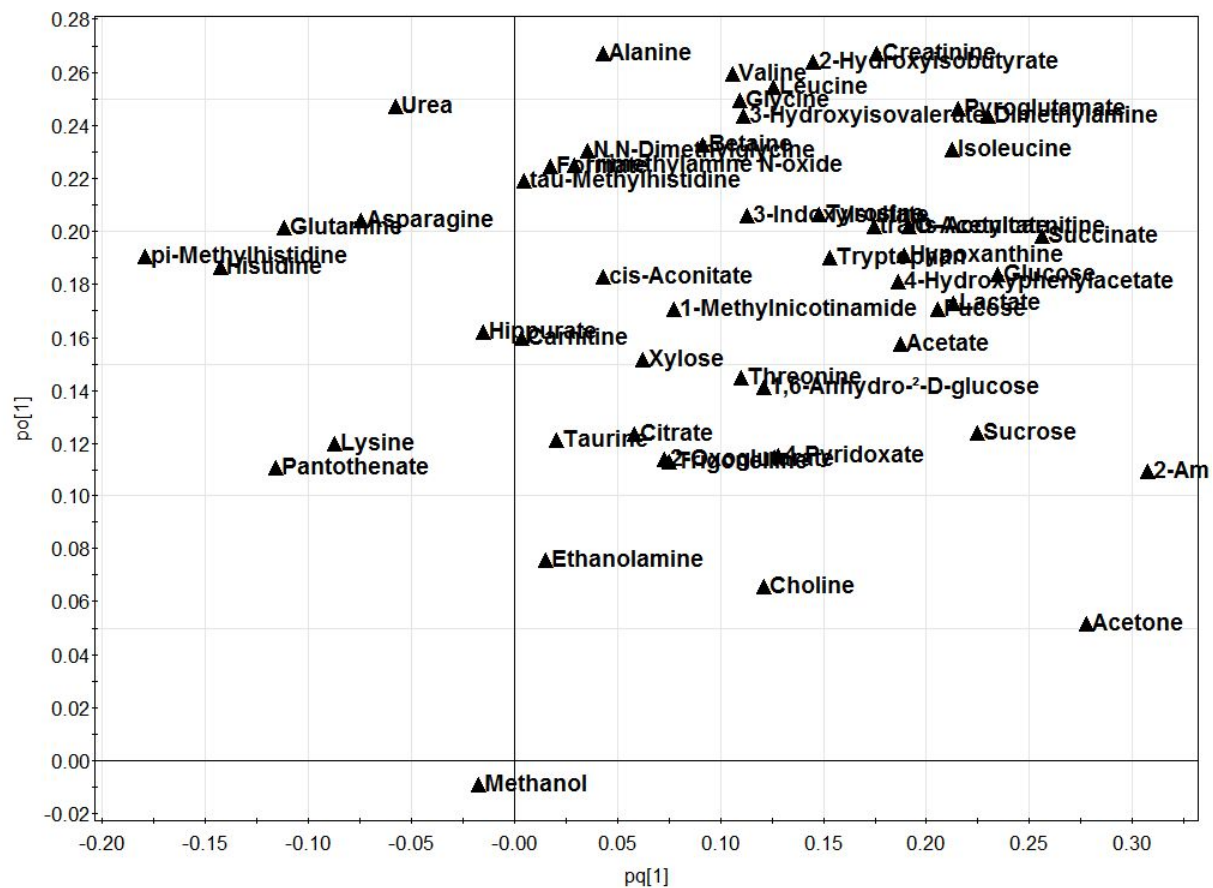

Supplement: Additional file 4 — OPLS-DA Loading Plot of Metabolite Profiles Derived From EAC and BE. [file 1477-7819-10-271-S4.pdf]

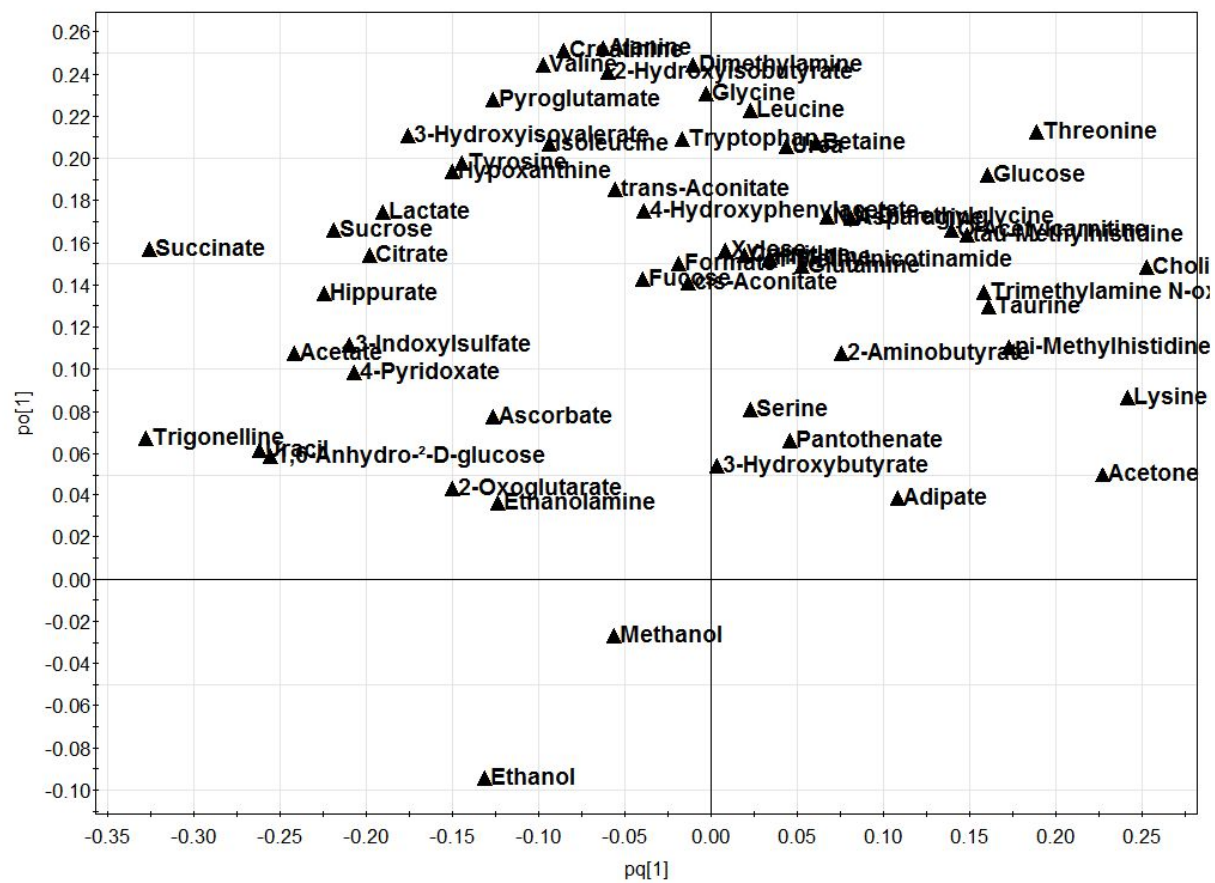

Supplement: Additional file 5 — OPLS-DA Loading Plot of Metabolite Profiles Derived From Esophageal and Pancreatic Cancer. [file 1477-7819-10-271-S5.pdf]

A)

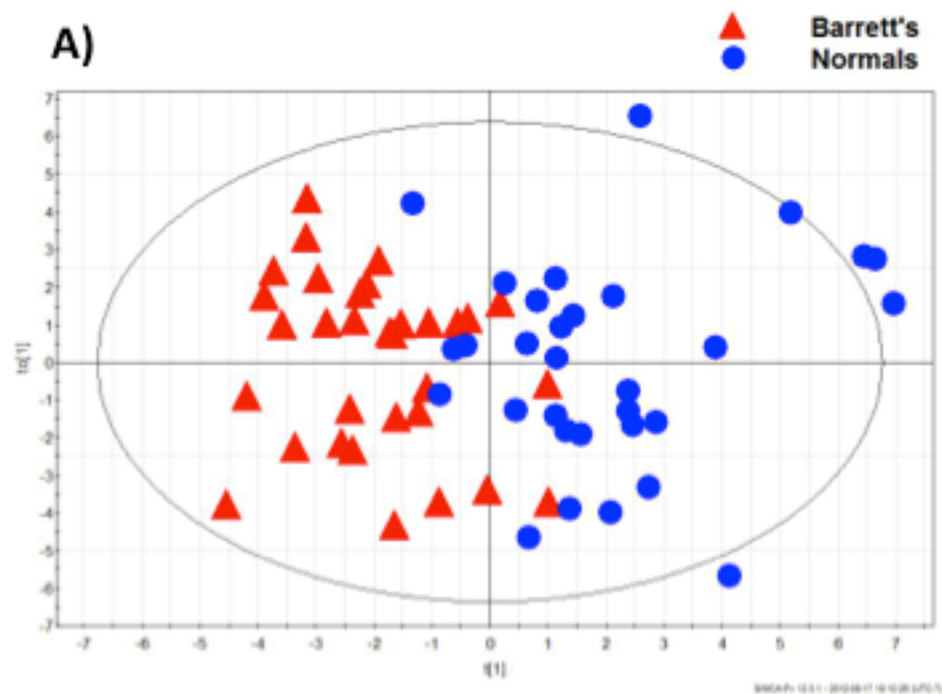

B)

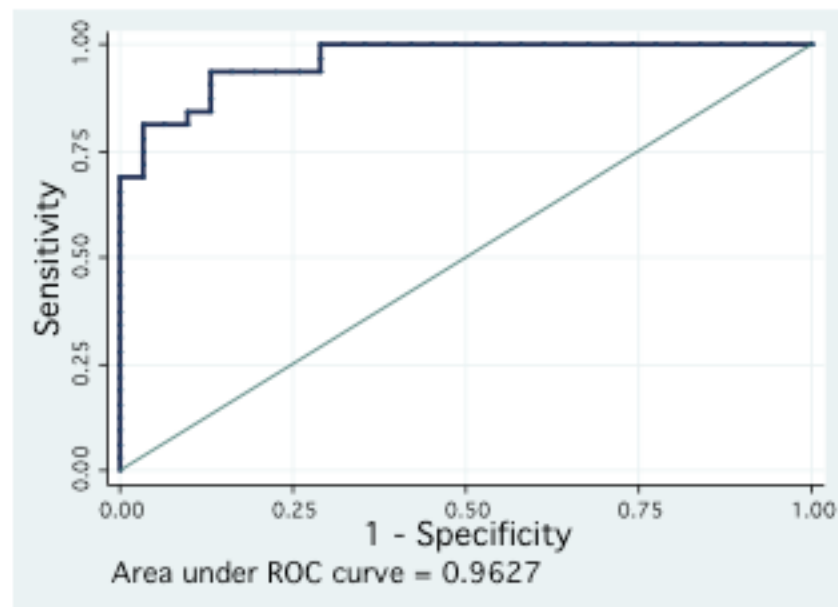

Supplement: Additional file 7 — OPLS-DA Score Plot of Metabolite Profiles Derived from BE and Healthy Controls with Corresponding ROC Curve Analysis. A) Supervised OPLS-DA score plot. Two-component model based on 53 measured urinary metabolites. BE is represented by red triangles and controls are depicted by blue circles. B) Corresponding ROC curve generated using cross-validated predicted-Y values of OPLS-DA model. AUROC = 0.9627. [file 1477-7819-10-271-S7.pdf]

A)

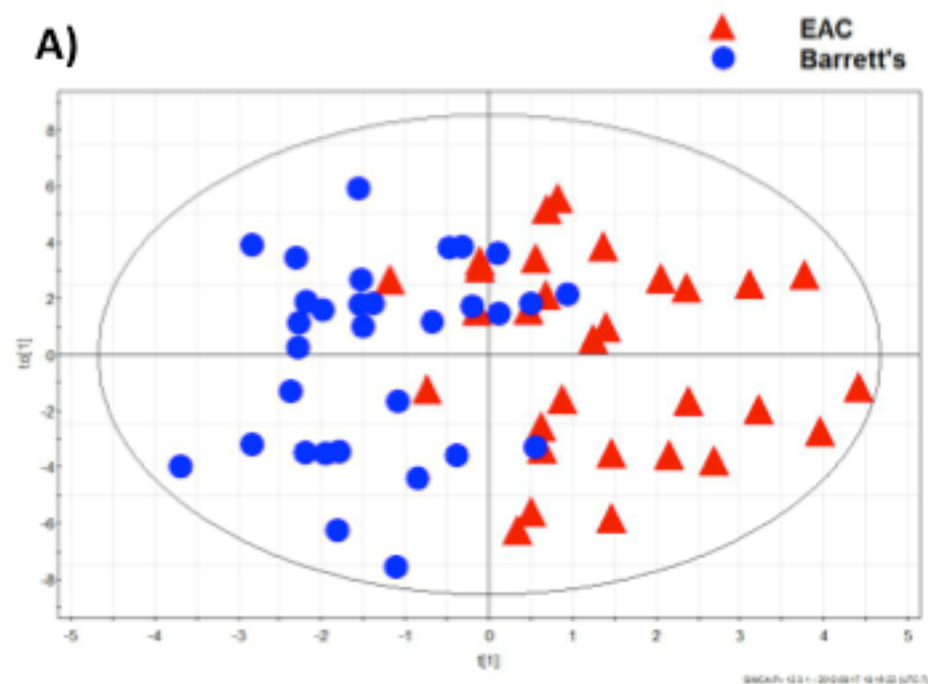

B)

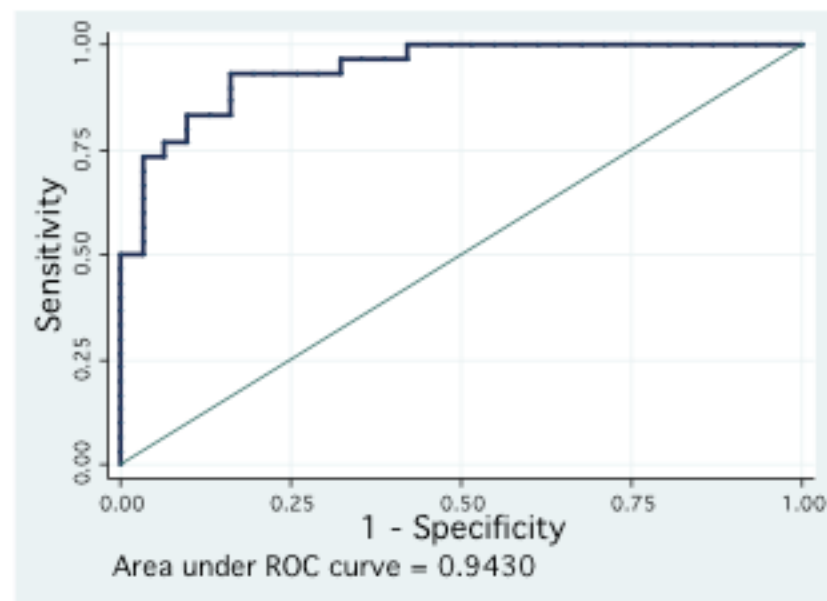

Supplement: Additional file 8 — OPLS-DA Score Plot of Metabolite Profiles Derived from EAC and BE with Corresponding ROC Curve Analysis. A) Supervised OPLS-DA score plot. Two-component model based on 53 measured urinary metabolites. BE is represented by blue circles triangles and EAC is depicted by red triangles. B) Corresponding ROC curve generated using cross-validated predicted-Y values of OPLS-DA model. AUROC = 0.9430. [file 1477-7819-10-271-S8.pdf]

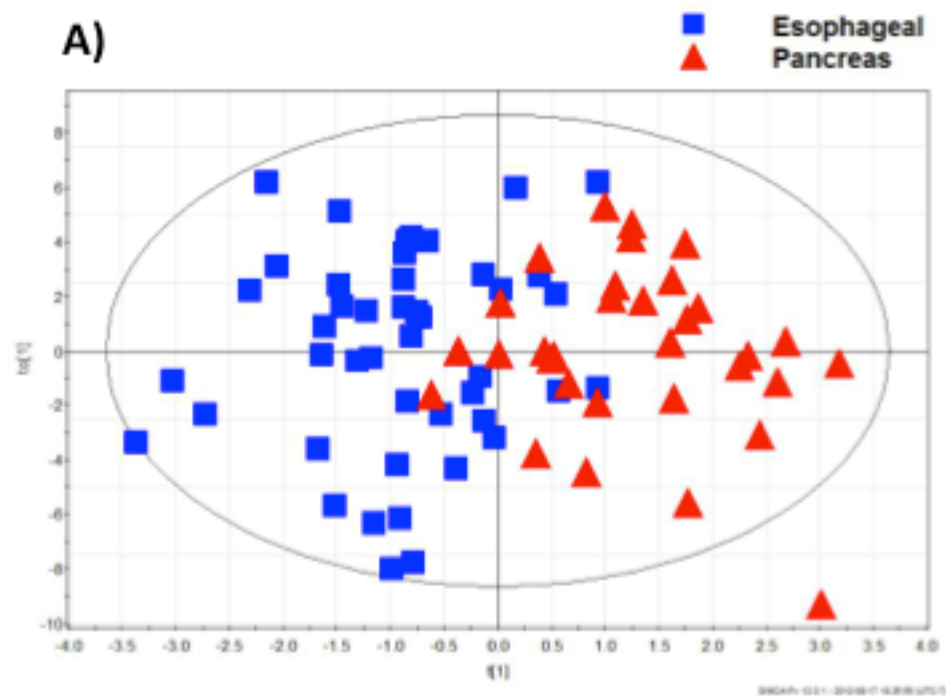

**B)**

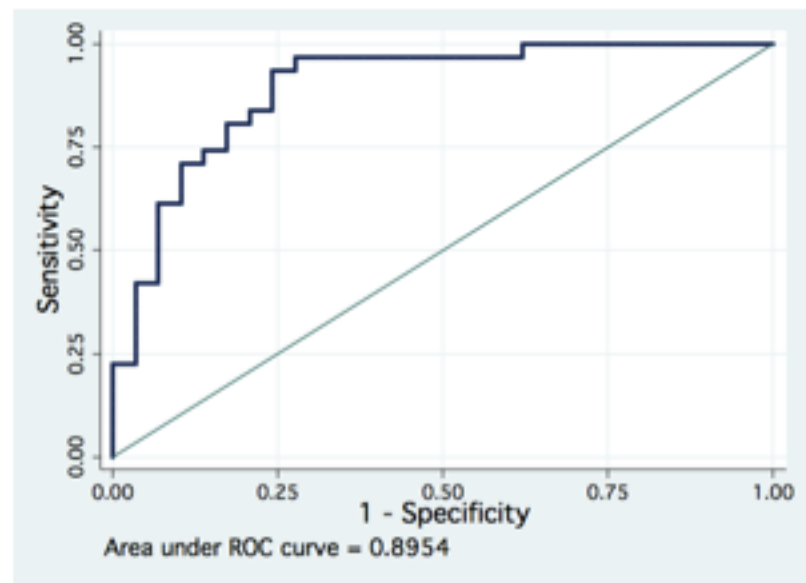

Supplement: Additional file 9 — OPLS-DA Score Plot Depicting Cancer Specificity of Urinary Metabolomic Profiles and Corresponding ROC Curve Analysis. A) Urinary metabolomic profiles of patients with esophageal carcinoma represented by blue squares, and pancreatic ductal adenocarcinoma, depicted by red triangles. B) Corresponding ROC curve generated using cross-validated predicted-Y values of OPLS-DA model. AUROC = 0.8954. [file 1477-7819-10-271-S9.pdf]
