# Supplementary material for: Urinary metabolomic signature of esophageal cancer and Barrett’s esophagus
Source: World J Surg Oncol. 2012 Dec 15;10:271. doi: 10.1186/1477-7819-10-271 (PMC3579706; doi:10.1186/1477-7819-10-271)
Supplement: Additional file 6 — Exogenous Metabolites with Source Description. [file 1477-7819-10-271-S6.pdf]

Exogenous Metabolites	Class	Source Description
**1,6 Anhydrogluclose**	*Carbohydrate*	Monosacccharide product of cellulose combustion excreted in urine<br>Potential dietary source includes caramel
**2-Hydroxyisobutyrate**	*Hydroxy acid*	Organic acid metabolite of methyl tert-butyl ether, obtained from environmental exposure
**4-Pyridoxate**	*Pyridoxals and derivatives*	Metabolic byproduct of vitamin B6 excreted in urine
**Adipate**	*Dicarboxylic acid*	Dicarboxylic acid often used as a gelling agent in dietary products
**Ascorbate**	*Hydroxy acid*	Essential nutritent of dietary origin. Vitamin C biologically active form
**Ethanol**	*Simple alcohol*	Alcohol present in biofludis from alcoholic consumtion<br>Small quantities produced from microbial metabolism and cleared with first pass metabolism
**Sucrose**	*Carbohydrate*	Disaccharide sugar used as a sweetner and found naturally in certain food plants
**Xylose**	*Carbohydrate*	Pentose sugar constituent of plant polysaccharides, present in legumes and some high fibre foods<br>Involved in carbohydrate metabolism
